# Clinical features and outcomes of invasive pneumococcal disease in a pediatric intensive care unit

**DOI:** 10.1186/s12887-015-0387-7

**Published:** 2015-07-17

**Authors:** Hsiang-Ju Hsiao, Chang-Teng Wu, Jing-Long Huang, Cheng-Hsun Chiu, Yhu-Chering Huang, Jainn-Jim Lin, I-Anne Huang, Oi-Wa Chan, I-Jun Chou, Shao-Hsuan Hsia

**Affiliations:** Department of Pediatrics, Chang Gung Memorial Hospital, Keelung, Taiwan; Chang Gung University College of Medicine, Taoyuan, Taiwan; Division of General Pediatrics, Department of Pediatrics, Chang Gung Memorial Hospital, Linkou, Taiwan; Division of Asthma, Allergy, and Rheumatology, Department of Pediatrics, Chang Gung Memorial Hospital, Linkou, Taiwan; Molecular Infectious Disease Research Center, Division of Pediatric Infection, Chang Gung Memorial Hospital, Linkou, Taiwan; Division of Pediatric Infectious Diseases, Department of Pediatrics, Chang Gung Memorial Hospital, Linkou, Taiwan; Division of Pediatric Critical Care Medicine, Department of Pediatrics, Chang Gung Memorial Hospital, Linkou, Taiwan

**Keywords:** Hemolytic uremic syndrome (HUS), herd immunity, invasive pneumococcal disease, mortality, pediatric intensive care unit, pneumococcal conjugate vaccine, serotype 19 F, *Streptococcus pneumoniae*

## Abstract

**Background:**

Invasive pneumococcal disease (IPD) results in high morbidity and mortality globally each year, although it is a vaccine-preventable disease. This study aimed to characterize the clinical features of IPD in a pediatric intensive care unit (PICU) in Taiwan. The seven-valent pneumococcal conjugate vaccine (PCV7) was introduced in the private sector in October 2005. The estimated coverage rate of PCV7 vaccination in 2010 was 45.5 % among children <5 years of age.

**Methods:**

We conducted a retrospective study at a single center in northern Taiwan for invasive pneumococcal disease in a PICU from 2009 to 2013. Demographic characteristics, clinical courses, serotype, antibiotic susceptibility, and outcomes were analyzed.

**Results:**

Over the 5-year study period, 2167 patients were admitted to the PICU; 48 (2.2 %) had IPD. There were 29 female and 19 male patients. Their mean age was 3.7 years (range 0.7–12.5 years, with the peak age at 2–5 years; *n* = 30, 63 %). Pneumonia was the most frequent type (*n* = 38, 79 %), followed by meningitis (*n* = 10, 21 %). In total, three patients died, all within 72 h after admission; the final diagnoses were all meningitis. Thirty-four children with pneumonia received chest tube insertion for pleural effusion drainage. Of them, 22 (65 %) finally still underwent video-assisted thoracoscopic surgery. Eight (17 %) children had hemolytic uremic syndrome, and seven of them underwent hemodialysis. In total, 37 serotypes were detected; 95 % were covered by PCV13. Serotype 19A was most common (54 %) overall; however, in those with meningitis, serotype 19 F was most common.

**Conclusions:**

Meningitis is the most severe type of invasive pneumococcal disease in our pediatric intensive care unit. It may progress rapidly even when subjects are given antibiotics promptly. The most common serotype in meningitis is 19 F, which is vaccine preventable. Thus, universal mass pneumococcal vaccination is still needed.

## Background

*Streptococcus pneumoniae* is a Gram-positive encapsulated bacterium. It can cause a wide disease spectrum, from otitis media to sepsis and meningitis [1]. Invasive pneumococcal disease (IPD) is defined as *S. pneumoniae* isolated from sterile sites, such as blood, cerebrospinal fluid, pleural fluid, or ascites. IPD results in high global morbidity and mortality each year [2], but it is a vaccine-preventable disease [3–5]. Its incidence decreased after the introduction of a seven-valent pneumococcal conjugate vaccine (PCV7) in the private sector in Taiwan in October 2005 [6, 7]. The estimated coverage rate of PCV7 vaccination was 5.7 % in 2006 and 45.5 % in 2010 among children <5 years of age. PCV13 was introduced in April 2010, and in 2013, a national catch-up program was launched in Taiwan to provide government-funded PCV13 for all children aged 2–5 years [7].

The symptoms and signs of IPD are related to host immunity, *S. pneumoniae* virulence, and the site of infection [1]. Early recognition and prompt diagnosis remain challenges. There have been few studies analyzing the early clinical presentation in severe IPD in children since the introduction of PCV7 in Taiwan. We designed this study to characterize the clinical features and outcomes of IPD in a pediatric intensive care unit (PICU) under partial PCV immunization.

## Methods

### Hospital setting

Chang Gung Memorial Hospital is a tertiary care hospital in northern Taiwan. Chang Gung Memorial Hospital is the only referral center in Taoyuan County, which has a population of 2 million people. The PICU is a 29-bed ICU serving children over 2 months of age. We reviewed the Chang Gung Memorial Hospital PICU patient database, which listed the name and diagnosis of every patient admitted to the PICU between January 2009 and December 2013. The study was approved by the institution review board (IRB: 100-2984A3).

### Clinical features

All children admitted to the PICU of Chang Gung Memorial Hospital with isolation of *S. pneumoniae* from sterile sites (blood, pleural fluid, and cerebrospinal fluid) were included. We collected data including demographics, underlying conditions or illnesses, previous hospitalizations, laboratory data, empiric and definite antimicrobial agents, requirements for and duration of intensive care, surgical intervention, final diagnosis, and outcome.

Pneumonia was defined as the presence of 1) radiological pneumonia plus positive blood culture, 2) *S. pneumoniae* isolates from pleural fluid culture, or 3) positive blood culture plus placement of chest tube for pleural fluid drainage. We defined meningitis as the presence of *Streptococcus pneumoniae* in cerebrospinal fluid or blood in association with a clinical diagnosis of meningitis [8].

The Glasgow Outcome Scale–Extended Pediatric Revision (GOS-E Peds) [9] was used for the assessment of outcome at 3 months after discharge. The GOS-E Peds uses the following eight-point scale: 1. upper good recovery, 2. lower good recovery, 3. upper moderate disability, 4. lower moderate disability, 5. upper severe disability, 6. lower severe disability, 7. vegetative state, and 8. death.

### Serotyping of *S. pneumoniae*

Detection of *S. pneumoniae* was first done in the Chang Gung Memorial or other hospital. Since October 2007, IPD has been listed as a notifiable disease in Taiwan [6]. It is mandatory to report IPD cases and for infection control personnel to deliver the isolated *S. pneumoniae* to the Centers for Disease Control, Taiwan (TCDC). Upon receipt, *S. pneumoniae* isolates were subcultured immediately and confirmed by standard microbiological methods, followed by serotyping using the capsular swelling method (Quellung reaction) [6]. The final report was faxed to Chang Gung Memorial Hospital.

### Statistical analysis

For analysis of serotype distribution, serotypes were categorized as PCV7 types (4, 6B, 9 V, 14, 18C, 19 F, and 23 F); PCV13 serotypes not included in PCV7 (1, 3, 5, 6A, 7 F, and 19A); and non-vaccine serotypes. Data analysis was performed using the SPSS software (ver. 21.0; SPSS, Chicago, IL). Categorical variables were compared using Fisher’s exact test. Descriptive statistics for continuous variables, such as laboratory parameters, were calculated and are reported as the means ± standard deviation (SD). The Mann–Whitney U-test was used to detect continuous variables differences between two groups. Statistical significance was set at *p* < 0.05.

## Results

### Epidemiology

Over the 5 years study period, 2167 patients were admitted to the PICU; of them, 48 (2.2 %) had IPD (Table 1). There were 29 female and 19 male patients; their mean age was 3.7 years (range, 0.7–12.5 years, with a peak in age 2–5 years (*n* = 30, 63 %). Pneumonia was the most frequent type (*n* = 38, 79 %), followed by meningitis (*n* = 10, 21 %). The mean PICU stay was 11.2 ± 11.9 days; the mean length of hospital stay was 21.6 ± 14.0 days. In total, three cases died, all died within 72 h after admission; the final diagnosis in all was meningitis. The case-fatality rate was 6.3 %. Most survived and recovered with no sequelae. However, there were four children with less than good recoveries (Table 2).

**Table 1:** Clinical features of IPDs in 48 children admitted to our pediatric intensive care unit

	Survival (*n* = 45)	Death (*n* = 3)	Total (*n* = 48)	*p*
Males	18	1	19	1.00
Age (years)				0.18
< 2	9	2	11	
2–5	29	1	30	
6–17	7	0	7	
Received vaccination	11	0	11	1.00
Symptoms and signs^1^				
Fever	45	3	48	
Cough and rhinorrhea	43	2	45	0.18
Vomiting without abdominal pain	15	3	18	0.05
Altered mental status	10	3	13	0.02
GCS <12	8	3	11	0.01
Seizure	9	3	12	0.01
Initial laboratory values				
WBC count (1000 cells/mm^3^)	14.7 ± 12.6	4.6 ± 2.1	14.1 ± 12.4	0.16
CRP (mg/L)	243 ± 107	178 ± 84	240 ± 107	0.32
Positive blood culture result	26	3	29	0.27
Disease category				0.01
Pneumonia	38	0	38	
Meningitis	7	3	10	
Length of hospital stay	22.9 ± 13.4	1.7 ± 1.2	21.6 ± 14.0	<0.01
in PICU	11.8 ± 12.1	1.7 ± 1.2	11.2 ± 11.9	<0.01

*WBC* white blood cells; *CRP* C-reactive protein

^1^All symptoms were at the time of presentation, except seizure, which was during the illness

**Table 2:** Details of pediatric IPD cases with less than good recoveries

Case	Age (years)	Gender	Initial presentation^1^	Clinical diagnosis	Serotype	Vaccinated	Outcome
1	1.1	M	Status epilepticus	Meningitis	6B	No	Lower severe disability
			GCS:E2V2M4				
2	6.2	M	Respiratory failure	Complicated pneumonia HUS	19 A	No	Upper moderate disability
3	0.7	M	Opitoshtonous	Meningitis	19 A	No	Lower severe disability
			GCS: E2V3M4				
4	1.9	F	Altered mental status	Meningitis	19 F	No	Death
			GCS: E1V1M2				
5	1.2	F	Altered mental status	Meningitis	23 F	No	Death
			GCS: E3V2M4				
6	9.4	F	Altered mental status	Meningitis + brain abscess	Not available	No	Vegetative state
			GCS: E2V2M4				
7	4.6	M	Altered mental status	Meningitis	19 F	No	Death
			GCS: E3V2M6				

^1^All patients had cough and rhinorrhea, with the exception of patients 1 and 5

*M* male; *F* female; *GCS* Glasgow Coma Scale; *HUS* hemolytic uremic syndrome

### Clinical characteristics

In total, 38 (79 %) patients initially presented with respiratory distress/failure, and all had radiological pneumonia; three of them presented initially with GCS ≤12. Another 10 (21 %) patients presented initially with altered mental status without radiological pneumonia and were finally diagnosed with meningitis; eight of them presented initially with GCS ≤12. Three children had no apparent preceding upper respiratory tract infection before admission; one of them died. Also, 12 children had seizures during the illness; 10 were diagnosed as having meningitis, and the other two as having septic shock with encephalopathy.

In total, 16 (33 %) patients needed intubation; six were ‘elective’ intubations to protect the airway because of altered mental status, and 10 were intubated because of respiratory failure. Also, 34 children with complicated pneumonia received chest tube insertion for pleural effusion drainage. Of them, 22 (65 %) ultimately underwent video-assisted thoracoscopic surgery (VATS). Eleven (23 %) patients (four meningitis and seven pneumonia) developed unstable hemodynamic status and had to be given inotropic agents, including dopamine, epinephrine, milrinone, or norepinephrine. One child (Patient 2, Table 2) had complicated pneumonia and developed respiratory failure with mechanical ventilation; this patient received extra-corporeal membrane oxygenation (ECMO) support. Unfortunately, he also had a stroke during his PICU stay, and had a moderate disability at follow-up. Also, eight (17 %) children had HUS; seven of them received hemodialysis. All were in the pneumonia group.

### Vaccination status, serotype, and antimicrobial resistance

The vaccination status of all children was known; 37 had not been vaccinated against IPD. A conjugated pneumococcal vaccine had been administered to 11 children prior to IPD; seven children received PCV7 and four received PCV13. In those who received PCV7, two children suffered from serotype 19A, a 4-year-old female who received one dose of PCV7 suffered from serotype 6A, and in four children, the serotypes were unknown. Of those who received PCV13, a 2-year-old female who received one dose of PCV13 suffered from serotype 6B, a 2-year-old male who received one dose of PCV13 suffered from serotype 19A, one child suffered from a non-typeable type, and in one child, the serotype was unknown. In the meningitis group, no patient had received a pneumococcal conjugated vaccine. However, at least 60 % of serotypes of the patients with meningitis were covered by PCV13 (Table 3).

**Table 3:** Demographic characteristics, serotype distribution, and outcomes in those with meningitis and pneumonia

	Meningitis*n* = 10	Pneumonia *n* = 38	*p*
Male	2	17	0.28
Age (years)			0.05
< 2	4	7	
2–5	3	27	
6–17	3	4	
Serotype distribution			
PCV7 serotypes			
6B	1	2	
14	0	5	
19 F	3	0	
23 F	1	1	
PCV13 serotypes not included in PCV7			
6A	0	1	
19A	1	19	
3	0	1	
Other	1^1^	1^2^	
Unknown	3	8	
Survival	7	38	0.01

^1^ 15,^2^non-typeable

In total, 37 serotypes were detected; 95 % were covered by PCV13 (Table 3). Serotype 19A was most common in our study. In 2009, serotype 14 was isolated in four children and serotype 19A in two children. In 2010 to 2013, only one serotype 14 cases was isolated; however, serotype 19A was isolated in 18 children. In 2012, serotype 15 was identified. In 2013, one non-typeable strain was identified. We further divided the patients into meningitis and pneumonia groups (Table 3); serotype 19 F was most common and was detected only in the meningitis group, and serotype 19A was most common type in pneumonia group. In those with HUS, serotype 14 was isolated in two children, 19A was isolated in three children, 6B was isolated in one child, and the serotype was not available in two children.

All patients were initially prescribed vancomycin plus ceftriaxone. Antimicrobial sensitivity testing was performed in 55 isolates. No resistance was detected against vancomycin. Only one *S. pneumoniae* with penicillin MIC <0.06 μg/mL was detected, and *S. pneumoniae* with penicillin MIC >2 μg/mL was detected in 20 % of all isolates. Three cases of *S. pneumoniae* with ceftriaxone MIC ≦0.05 μg/mL were detected, and *S. pneumoniae* with ceftriaxone MIC ≧2 μg/mL was detected in 16 % of all isolates; two were isolated from CSF.

### Outcomes

Three patients died in this study; all had meningitis. The prognostic factors for mortality were GCS < 12 (*p* = 0.01), seizure (*p* = 0.01), and vomiting without abdominal pain (*p* = 0.05). All of these three patients had the same characteristics; therefore, the data did not permit the use of logistic regression due to the lack of “complete separation.” Gender, headache, white cell counts, C-reactive protein, and vaccination status were not significantly associated with mortality. Children without good recovery (Table 2) were more frequent in children <2 years (4/11, 36 %) than those between 2 and 5 years old (1/30, 3 %) or those 6–17 years (2/7, 33 %; *p* = 0.01).

## Discussion

We provide the first detailed description of the severe IPD in a PICU under partial PCV immunization in northern Taiwan. In our study, we examined a series of 48 patients diagnosed with IPD at a tertiary hospital in Taiwan. Patients with IPD had two distinct types of presentation: 79 % (38/48) had complicated/uncomplicated pneumonia, presenting as respiratory distress/failure, and the most common serotype in pneumonia was 19A. The other 21 % (10/48) children had meningitis, presenting as altered mental status. Of them, three children died within 72 h, and 19 F was the most common serotype in meningitis. All three children who died had the same characteristics, namely GCS <12, seizure, and vomiting without abdominal pain.

Pneumonia was the most common type of IPD in our series. Our study, like others [3, 6, 10, 11], highlights the prevalence of serotype 19A after PCV7 was introduced. This may have been the result of vaccine selection. PCV7 was introduced to Taiwan in 2005, and the estimated coverage rate of PCV7 vaccination was 45.5 % in 2010 among children <5 years of age. [7]. For those with pneumonia, all patients showed good recovery except one child who had moderate disability after dialysis and ECMO. This may be the result of advances in critical care medicine over the past decade [12]. Hemolytic uremic syndrome is one of the most common complications of necrotizing pneumonia in Taiwan [13–16]. In our study, seven patients received hemodialysis because of HUS. In North America, 50 % of pneumococcus HUS was associated with serotype 19A after the introduction of PCV 7 [12, 17]; our study showed similar results.

Meningitis was the second most frequent type of IPD in our study; however, all three children who died had meningitis. Among those with meningitis, no *S. pneumoniae* penicillin MIC <0.06 μg/mL was isolated, and 20 % were resistant to Ceftriaxone . Thus, in those with suspected meningitis, empiric antibiotics, such as vancomycin plus a third-generation cephalosporin, are suggested [18]. We prescribed vancomycin plus ceftriaxone initially, yet three children still died within 72 h. Kanegaye et al. reported that 4 h after parental antibiotics, cerebrospinal fluid sterilization of pneumococcus might occur [19]. These findings indicate that the damage caused by *S. pneumoniae* infections may not be stopped by eradicating *S. pneumoniae*.

The three children who died had the same characteristics, namely seizures, GCS <12, and vomiting without abdominal pain; all of these signs indicate increased intracranial pressure (IICP). *S. pneumoniae* not only results in disruption of the blood brain barrier but also in neuronal and vascular injury, finally leading to IICP and diffuse brain ischemia [20, 21]. Early antibiotic use is essential; aggressive control of IICP is also important. Adjunctive therapy is an important issue for pneumococcal meningitis [22], patients with meningitis due to *S. pneumoniae* who were treated with corticosteroids had a lower death rate [23].

Serotype 19 F was the most common type in the meningitis group in our study. In adults, serotype 19 F has been associated with an elevated risk of meningitis and death [24]. Hsu et al. reported the effects of a pneumococcal conjugate vaccine on pneumococcal meningitis; it showed that serotypes 9 V, 14, and 23 F had better herd immunity than serotypes 4, 6B, 18C, and 19 F [3]. Wei et al. also reported that for all serotypes included in PCV, except serotype 19 F, demonstrated a sustained decreasing trend between 2008 and 2014 in Taiwan [7]. In our study, at least 60 % of the meningitis serotypes were vaccine-preventable. None of the children had an underlying illness; other studies have also shown that most children with IPD were previously healthy [7]. Thus, universal mass pneumococcal vaccination is still needed.

Being aged less than 2 years was not a predictor of mortality, but it may predict those who will not have a good recovery [10]. In 2013, a national catch-up program was launched in Taiwan to provide government-funded PCV13 for children aged 2-5 years [7]. In 2014, the Taiwanese government expanded the age group of children to 1 year of age. In Taiwan, the prevalence of *S. pneumoniae* colonization in children was 14.1 %. By breaking the chain of disease transmission and reducing the nasopharyngeal carriage rate among children, this policy may help to protect children less than 1 year old.

This study had some limitations. First, this was a retrospective, single-center study, and the sample size was limited. Additionally, the three patients who died had the same characteristics, so our data could not support logistic regression analysis due to the absence of “complete separation”. Second, some patients had received antibiotics before admission; these resulted in false-negative culture results, and some true IPD cases were likely not included. Polymerase chain reaction (PCR) would improve diagnostic yield [10]. Third, some *S. pneumoniae* serotypes were not available. Taiwan CDC provides the *S. pneumoniae* serotype only to the first hospital reporting IPD; thus, cooperation between hospitals is needed. Fourth, host immunity is an important factor for IPD; in Taiwan, the rate of asymptomatic carriage of *S. pneumoniae* in children aged 2–5 years was 19 % [25], and the incidence of IPD among children aged 2–4 years was 21.1 per 100,000 populations, strongly suggesting that host immunity plays a key role. Gaschignard et al. suggested that children with IPD, particularly those aged >2 years, should undergo immunological investigation [26]. We have been performing immunological investigations since 2012, and we will report those results in future.

## Conclusions

Our study showed a wide spectrum of invasive pneumococcal disease in our pediatric intensive care unit. Meningitis is the most severe type. Meningitis can progress rapidly even when subjects are given antibiotics promptly. The most common serotype in meningitis was 19 F, which is vaccine preventable. Thus, universal mass pneumococcal vaccination is still needed. We observed replacement of pneumococcal serotypes, specifically, the prevalence of serotype 19A. Further study on controlling increased intracranial pressure caused by *S. pneumoniae* meningitis is needed.

## Consent

The study was approved by the institution review board (IRB: 100-2984A3).

## Abbreviations

ECMO: extra-corporeal membrane oxygenation
GCS: Glasgow Coma Scale
GOS-E Peds: Glasgow Outcome Scale–Extended Pediatric Revision
HUS: hemolytic uremic syndrome
IICP: increased intracranial pressure
IPD: invasive pneumococcal disease
MIC: minimum inhibitory concentration
PCV: pneumococcal conjugate vaccine
PCV7: seven-valent pneumococcal conjugate vaccine
PCV13: 13-valent pneumococcal conjugate vaccine
PICU: pediatric intensive care unit
VATS: video-assisted thoracoscopic surgery
